# Multi-country clinical practice patterns, including use of biomarkers, among physicians’ treatment of BCG-unresponsive non-muscle invasive bladder cancer (NMIBC)

**DOI:** 10.1186/s12894-022-00959-z

**Published:** 2022-02-26

**Authors:** Edward I. Broughton, Kyna M. Gooden, Katie L. Mycock, Ivana Rajkovic, Gavin Taylor-Stokes

**Affiliations:** 1 Bristol Myers Squibb, Lawrence Township, NJ USA; 2 Adelphi Real World, Bollington, UK

**Keywords:** Urinary bladder neoplasms, Non-muscle invasive bladder cancer, Biomarkers, Disease management, Clinical practice patterns, Immunotherapy, Real-world evidence

## Abstract

**Background:**

Intravesical bacillus Calmette-Guérin (BCG) fails in a considerable proportion of non-muscle invasive bladder cancer (NMIBC) patients despite treatment per recommended protocol. This real-world study aimed to understand the current patterns of treatment and disease management for the broad BCG-unresponsive NMIBC patient population, alongside collecting sufficient data on patients who do not undergo cystectomy.

**Methods:**

This was a multicenter, retrospective survey of physicians treating BCG-unresponsive NMIBC patients. Data were collected in eight countries – France, Germany, Spain, Italy, United Kingdom, United States, China, and Japan – between January and May 2019. The study consisted of a short online physician survey and a retrospective chart review of eligible BCG-unresponsive NMIBC patients. Physicians abstracted chart data for the last 10 (five patients in Japan) eligible BCG-unresponsive NMIBC patients meeting the inclusion criteria, and the data were analysed for all countries combined using descriptive statistics. Country-specific analyses were also carried out, as appropriate.

**Results:**

Overall, 508 physicians participated in the study. Almost one-quarter (22.9%) of physicians’ current NMIBC patient caseload was BCG-unresponsive, whereby BCG therapy was no longer considered an option. Half of physicians (49.4%) did not regularly use biomarker tests in their practice, with particularly few physicians undertaking biomarker testing in Spain and Japan. Biomarker testing varied considerably, with the proportions of physicians selecting ‘none’ ranging from 11.4% in China to 70.3% in Japan. Physicians reported transurethral resection of the bladder tumor (TURBT) and BCG as the most common current treatments received by their patients. Chemotherapy and anti-PD-L1 treatment options were considered impactful new therapies by 94.7% and 90.0% of physicians surveyed in this study, respectively.

**Conclusions:**

The most common treatments received by patients in this study were TURBT and BCG. Emerging new treatments are driven by exploring biomarkers, but in real-world clinical practice only half of physicians or fewer regularly tested their NMIBC patients for biomarkers; PD-1/PD-L1 was the most common biomarker test used. Most physicians reported that, in addition to chemotherapy, anti-PD-L1 was an impactful new therapy.

**Supplementary Information:**

The online version contains supplementary material available at 10.1186/s12894-022-00959-z.

## Background

Bladder cancer is the tenth most common cancer worldwide [1], with an estimated 573,278 newly diagnosed cases and 212,536 deaths in 2020. Bladder cancer is classified as non-muscle invasive bladder cancer (NMIBC) or muscle invasive bladder cancer (MIBC), depending on the presence or absence of invasion of the primary tumor into the muscle wall of the bladder [2]. Following diagnosis and tumor staging, NMIBC tumors are stratified as ‘low-’, ‘intermediate-’, or ‘high-risk’ according to risk of recurrence and/or progression, based on the European Organization for Research and Treatment of Cancer (EORTC) scoring tables [2]. Patients in the ‘high-risk’ group have an increased 5-year risk of recurrence (up to 78%) and progression (up to 45%) according to the EORTC risk stratification tables [3].

Current standard of care (SOC) for ‘intermediate- ‘ and ‘high-risk’ NMIBC is transurethral resection of the bladder tumor (TURBT) followed by intravesical therapy [4], most commonly with intravesical bacillus Calmette-Guérin (BCG) – the latter successfully delays tumor recurrence and disease progression but fails in up to 50% of this population despite treatment per recommended protocol, with around half of failures usually occurring within the first six months [4]. Moreover, it has been reported that tumors recur in 30 − 40% of NMIBC cases, and around 20% of ‘high-risk’ cases initially treated with BCG eventually progress to MIBC [5].

Following SOC failure, current guidelines recommend radical cystectomy for ‘high-risk’ NMIBC [4, 6, 7]. However, this procedure is associated with increased risk of morbidity and mortality and impaired quality of life (QoL) [4, 6, 8, 9], and a notable subset of patients refuse cystectomy or are not suitable surgical candidates [4, 7, 10–13]. As a result, there is a significant unmet need for the development of effective non-surgical treatments for BCG-unresponsive NMIBC patients [7, 13, 14]. Since 1959, the Food and Drug Administration (FDA) has only approved two additional intravesical therapies for bladder cancer – valrubicin and thiotepa – and neither has demonstrated robust efficacy [11]. However, other strategies are currently being investigated in BCG-unresponsive NMIBC, including several novel immunotherapy strategies, such as immune checkpoint inhibitors targeting the PD-1/PD-L1 axis (e.g. pembrolizumab, atezolizumab, durvalumab, nivolumab), small molecule inhibitors of indoleamine (2,3)-dioxygenase 1 (linrodostat mesylate, epacadostat), an interleukin-15 superagonist (ALT-803), viral gene therapies (Adstiladrin, CG0070), and vaccines (PANVAC) [4]; exploratory biomarker analyses may in future help identify those patients most likely to respond to specific therapies or mechanisms of action [4].

As there is little evidence on the current treatment patterns of patients with BCG-unresponsive NMIBC, the overall primary objective of the current study, which was undertaken in North America, Europe, and Asia, was to understand the current patterns of treatment and disease management for the broad BCG-unresponsive NMIBC patient population, alongside collecting sufficient data on patients who do not undergo cystectomy (surgery).

## Methods

### Study design and data collection

This was a multicenter, retrospective survey of physicians treating BCG-unresponsive NMIBC patients (including urologists, uro-oncologists, and medical/clinical oncologists). Data were collected in eight countries – France, Germany, Italy, Spain, United Kingdom (UK), United States (US), China, and Japan – between January and May 2019. The study examined real-world data on the use of biomarkers in NMIBC patients for the diagnosis of BCG-unresponsiveness and for surveillance and treatment selection in clinical practice: initial treatment, surveillance, and subsequent treatment. It consisted of a short online physician survey (survey questions are included in Additional file 1:  Table S2) and a retrospective chart review of eligible BCG-unresponsive NMIBC patients. The latter was completed for a broad cohort of BCG-unresponsive patients who may/may not have undergone cystectomy (Cohort 1), as well as for an enriched sample of patients who were BCG-unresponsive and did not undergo cystectomy (Cohort 2), either because the patient was medically ineligible for a cystectomy (based on their physician’s assessment) or because they had refused cystectomy.

Physicians abstracted chart data for the last 10 (five patients in Japan) eligible BCG-unresponsive NMIBC patients meeting the inclusion criteria (listed below): the last five patients (two patients in Japan) seen by the physician from the broad BCG-unresponsive population, then the last five patients (three in Japan) defined as BCG-unresponsive, prior to Cohort 1, who met the inclusion criteria and did not undergo radical cystectomy (ineligible/refuse surgery) for the enriched population. Japanese physicians provided data for up to 5 eligible patients, based on feasibility assessments in the design phase and the physician universe size—this sample size was determined as most appropriate.

For the purposes of the present study, BCG-unresponsive NMIBC was defined as at least one of the following (FDA definition, 2018) [15]:

1. Persistent or recurrent carcinoma in situ (CIS) alone or with recurrent Ta (non-invasive papillary carcinoma)/T1 (invasion limited to the lamina propria) disease within 12 months of completion of adequate BCG therapy;
  a. Adequate BCG therapy was defined as at least one of the following: at least five of six doses of an initial induction course, plus at least two of three doses of maintenance therapy; at least five of six doses of an initial induction course plus at least two of six doses of a second induction course);
2. Recurrent high-grade Ta/T1 disease within six months of completion of adequate BCG therapy;
3. T1 high-grade disease at the first evaluation following an induction BCG course [16].

Participating physicians were identified via local data collection partners and were invited to participate in the study following completion of a short screening questionnaire. Only physicians who saw a minimum number of patients (10 in all countries, except Japan, where four was minimum) were included in the study. Physicians were required to have qualified as a physician 2 − 35 years prior to data collection; be actively involved in the management of NMIBC patients who were BCG-unresponsive (for a minimum of three months prior to the date of data abstraction; for the enriched sample, physicians were also required to manage patients who did not undergo cystectomy [ineligible/refused patients]); perform/offer radical cystectomy as a part of standard practice; and be able to provide a minimum of 10 patient records for the defined patient cohorts (five patient records in Japan).

Participating patients in both cohorts were required to be aged ≥ 18 years; have a physician confirmed diagnosis of NMIBC; and be classified as BCG-unresponsive for a minimum of three months prior to the start of data abstraction.

Data collection for this study was conducted in accordance with the guidelines provided by the European Pharmaceutical Market Research Association and aligned domestic organizations. The study was conducted in accordance with the Health Insurance Portability and Accountability Act guidelines, and no patient identities were collected. All data were de-identified and aggregated.

### Analysis

The data were analysed for all countries combined (i.e., aggregated global data), using descriptive statistics, and results were interpreted at a global level. In addition, country-specific analyses were carried out, as appropriate, to highlight differences between individual countries or regions.

Respondents were required to answer each question before they were able to complete the next and therefore there were no missing data, as ‘don’t know’ or ‘unknown’ were valid responses.

Analyses were performed in IBM® SPSS® Data Collection Survey Reporter v7.5 software, with statistical analyses conducted in Stata statistical software version 16.0 or later [17].

## Results

Overall, 508 physicians (France: 39; Germany: 39; Italy: 39; Spain: 49; UK: 36; US: 180; China: 35; Japan: 91) completed a physician survey and an electronic case report form (CRF) for the last 10 (five in Japan) qualifying NMIBC patients (which provided a total of 2554 CRFs (France: 304; Germany: 300; Italy: 301; Spain: 301; UK: 300; US: 600; China: 303; Japan: 145).

### Physician demographics and caseload

Most physicians were urologists (n = 287; 56.5%) or medical oncologists (n = 139; 27.4%). Additional demographics are included in Additional file 1:  Table S1. Of physicians’ total bladder cancer caseload, 38 (28.4%) were MIBC, and 96 (71.6%) were NMIBC cases. Physicians (n = 508) stated that 22.9% of their total NMIBC patient caseload was BCG-unresponsive, and BCG was therefore no longer a treatment option. However, they also stated that 37.7% of their NMIBC patient caseload had not received any BCG treatment for NMIBC (this ranged from 20.4% in China to 66.8% in Japan).

Of all (n = 508) physicians’ current (i.e., at the time of data collection) BCG-unresponsive caseload, 56.6% of cases were considered ‘high-risk’ and 43.4% were considered ‘intermediate-risk’. More than half (n = 278; 54.7%) of physicians stated that they followed national guidelines when treating NMIBC patients. Other guidelines that were used by large proportions of physicians were National Comprehensive Cancer Network (n = 252; 49.6%), American Urological Association/Society of Urologic Oncology (n = 244; 48.0%), and European Association of Urology (n = 218; 42.9%).

### Patient demographics and clinical characteristics

Patient demographics and characteristics are summarised in Table 1.

**Table 1:** Patient demographics and clinical characteristics

Demographics and clinical characteristics	n = 2554
Age (years), mean (SD)	71.5 (9.9)
Gender (male), n (%)	1978 (77.4)
BMI, mean (SD)	25.9 (4.5)
Risk classification at diagnosis, n (%)	
High	1248 (48.9)
Intermediate	1306 (51.2)
ECOG at initial NMIBC diagnosis, n (%)	
0	1209 (47.3)
1	805 (31.5)
≥ 2	488 (19.0)
Unknown/not assessed	52 (2.0)
Number of tumors at most recent assessment, n (%)	
1	963 (37.7)
2 − 7	1419 (55.6)
> 8	172 (6.7)
Time since diagnosis (years), mean (SD)	1.9 (2.5)
Staging at diagnosis, n (%)	
CIS alone	871 (34.1)
Papillary alone	859 (33.6)
Papillary + CIS	824 (32.3)

BMI: body mass index; CIS: carcinoma in situ; ECOG: Eastern Cooperative Oncology Group; NMIBC: non-muscle invasive bladder cancer; SD: standard deviation

### Use of biomarkers

#### For diagnosis

**Fig. 1:**
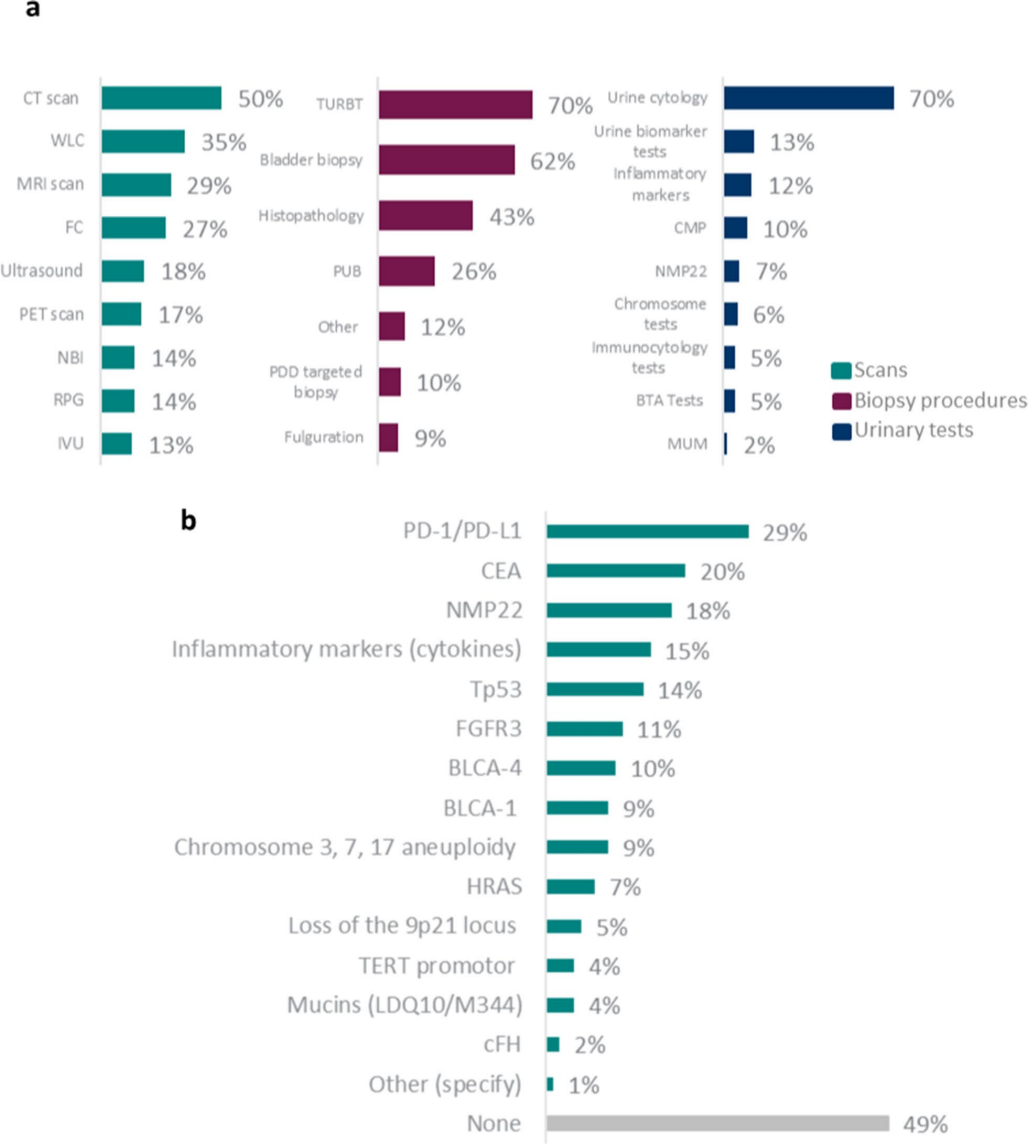
Physicians’ (n = 508) techniques for diagnosing NMIBC BCG-unresponsiveness (**a**) and biomarker tests in NMIBC patients (**b**)

To determine if a patient was BCG-unresponsive, almost all physicians (n = 501; 98.6%) reported that they used scans/biopsy procedures/urinary tests. Half (n = 252; 49.6%) of the physicians surveyed reported that they used computerised tomography scans, 70.1% (n = 356) used TURBT, and 70.3% (n = 357) used urinary tests (Fig. 1a).

#### For surveillance

Half of physicians (n = 251; 49.4%) reported that they did not regularly test for biomarkers in NMIBC patients (Fig. 1b). Country-specific analysis revealed that lack of regular testing for biomarkers in NMIBC patients was particularly high in Spain (49 physicians; 67.3%) and Japan (91 physicians; 70.3%) (Table 2). Where tested, 29.3% (n = 149) of physicians reported that they regularly tested for PD-1/PD-L1 (Fig. 1b). PD-1/PD-L1 testing among physicians was greater than half in China and Germany; approximately one-third in France, Italy, UK, and US; and less than one-fifth in Spain and Japan. Regular carcinoembryonic antigen testing was particularly high in China (35 physicians; 82.9%) and Italy (39 physicians; 51.3%) compared with other countries (range 3.3 − 20.5% of physicians) (Table 2). A total of 717 patients (28.1%) were tested for PD-L1 and of these, over one-third (n = 244; 34.0%) were positive, 283 (39.5%) were negative, 135 (18.8%) had an inconclusive result, and 55 (7.7%) were either awaiting results or their result was unknown. On average, the PD-L1 expression in these patients was 21.1%. PD-L1 expression was high in Asia, with a particularly high percentage in China (99 patients; 62.6%).

**Table 2:** Country-specific proportions of physicians who regularly tested for biomarkers in NMIBC patients

Biomarker	FR (n = 39)	DE (n = 39)	IT (n = 39)	ES (n = 49)	UK (n = 36)	US (n = 180)	JP (n = 91)	CN (n = 35)
PD-1/PD-L1	41%	56%	38%	18%	39%	27%	7%	54%
CEA	18%	21%	51%	12%	4%	14%	3%	83%
NMP22	5%	13%	3%	4%	3%	24%	21%	20%
Inflammatory markers†	5%	13%	21%	4%	2%	13%	1%	51%
Tp53	10%	13%	15%	4%	1%	18%	2%	31%
FGFR3	13%	15%	5%	6%	5%	1%	2%	9%
BLCA-4	8%	5%	5%	6%	19%	1%	1%	20%
BLCA-1	10%	8%	5%	4%	5%	12%	3%	23%
Chromosome aneuploidy‡	3%	3%	3%	0%	1%	16%	1%	20%
HRAS	0%	5%	5%	4%	1%	9%	3%	11%
Loss of 9p21 locus	3%	8%	3%	2%	3%	8%	8%	0%
TERT promoter	0%	5%	5%	0%	3%	6%	2%	6%
Mucins (LDQ10/M344)	0%	3%	0%	2%	1%	5%	1%	9%
cFH	3%	3%	0%	0%	3%	3%	3%	3%
Other	3%	0%	3%	2%	3%	1%	0%	0%
None	49%	38%	31%	67%	33%	51%	70%	11%

CEA: carcinoembryonic antigen; †Inflammatory markers (cytokines); ‡Chromosome 3, 7, 17 aneuploidy

CN: China; DE: Germany; ES: Spain; FR: France; IT: Italy; JP: Japan; UK: United Kingdom; US: United States

### Treatment selection

#### Initial treatment

Physicians reported that they took into consideration several factors to guide initial treatment decisions for NMIBC patients. Most physicians considered ‘tumor stage’ (n = 430; 84.6%) and ‘tumor grade’ (n = 434; 85.4%), closely followed by the presence of CIS (n = 405; 79.7%) and recurrence history (n = 404; 79.5%%).

#### Surveillance

**Fig. 2:**
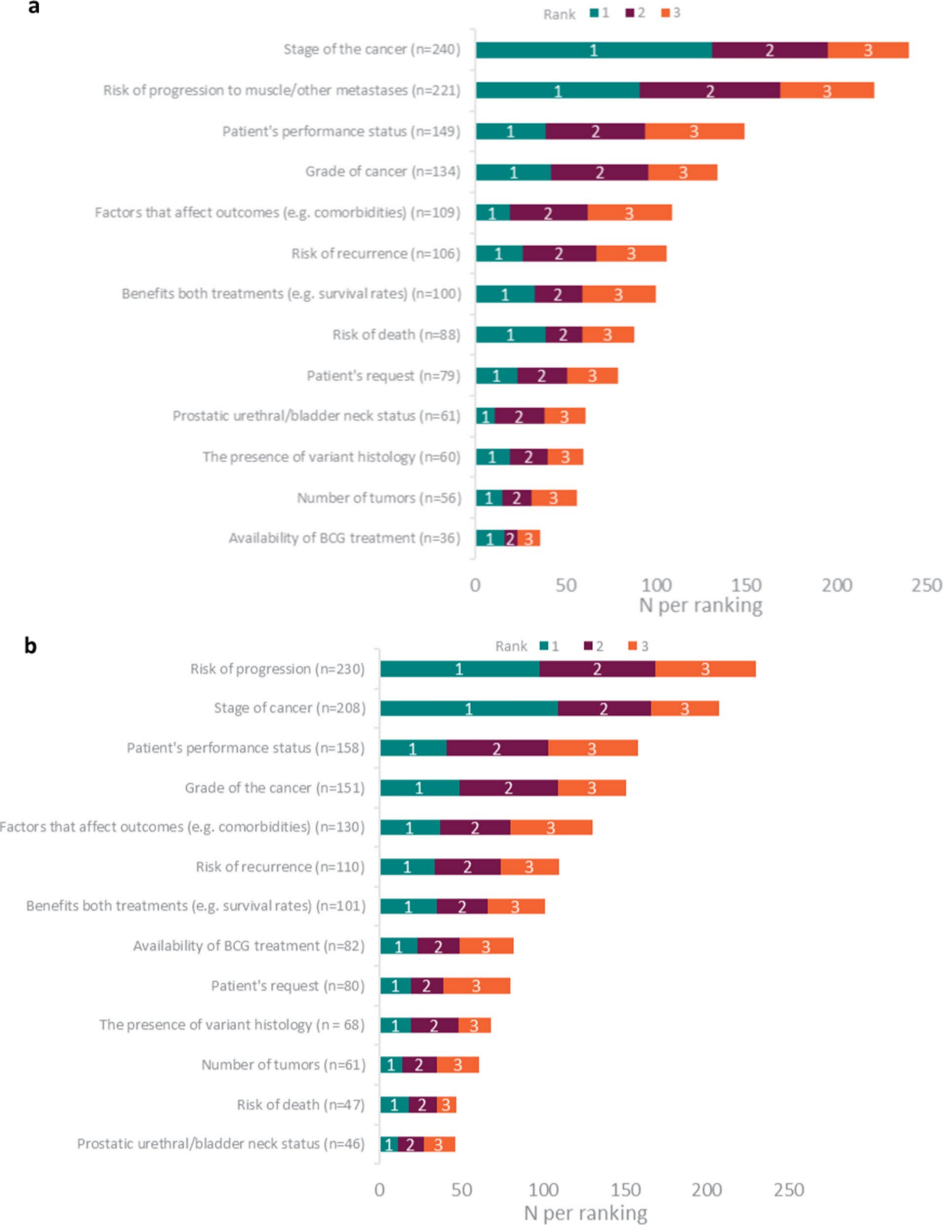
Physicians’ (n = 508) considerations when recommending radical cystectomy over BCG treatment (**a**), and vice versa (**b**)

Physicians also reported that they considered several different factors when recommending radical cystectomy in preference to BCG treatment (Fig. 2a). Most physicians considered ‘stage of cancer’ (330 physicians; 65.0%) and ‘risk of progression’ (to muscle or other metastases) (307 physicians; 60.4%). ‘Stage of cancer’ was ranked as the most important consideration overall (Rank 1), followed by ‘risk of progression’.

Similarly, physicians considered several different factors when recommending BCG treatment in preference to radical cystectomy (Fig. 2b). Most physicians considered ‘risk of progression’ (355 physicians; 69.9%), ‘patient’s performance status’ (328 physicians; 64.6%), ‘stage’ (332 physicians, 65.4%) and ‘grade’ (325 physicians; 64.0%) of cancer.

### Treatment effectiveness – physician attitudes (current management approach)

**Fig. 3:**
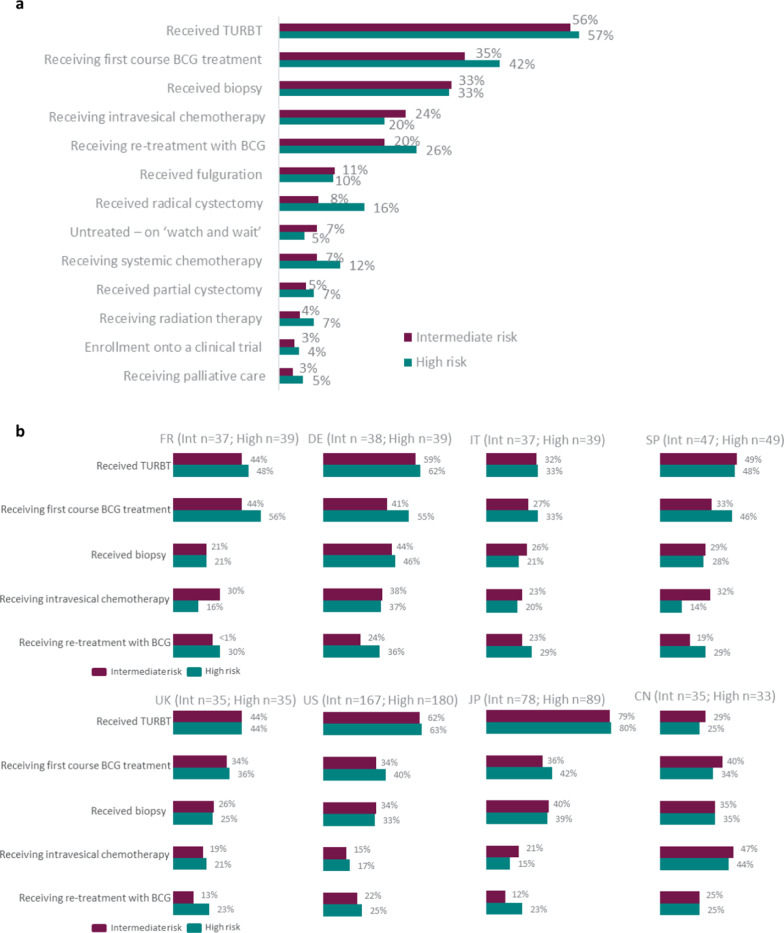
**a**,** b** Physician proportions (n = 508) reporting current management approaches for NMIBC patients, split by ‘intermediate-‘ and ‘high-risk’

Physicians reported that over half of all their NMIBC patients (‘intermediate-risk’ [n = 503; 57.3%] and ‘high-risk’ [n = 474; 55.6%]) were receiving TURBT at the time of data collection (Fig. 3a). Physicians treating ‘intermediate-risk’ group patients (n = 474) reported that 35.4% and 20.2% of patients were receiving first course or re-treatment with BCG, respectively. Physicians treating ‘high-risk’ group patients (n = 503) reported that the equivalent numbers were 42.2% and 26.3%. Higher reports of radical cystectomy were observed in the ‘high-risk’ group in Germany (n = 39; 24.4%), Italy (n = 39; 23.2%), Spain (n = 49; 25.1%), and China (n = 33; 23.5%) compared with the other countries (range 9.8 − 15.0%). The same was observed in China vs. other countries for partial cystectomy (n = 33; 21.8% vs. range 0.9 − 11.7%). In addition, the use of systemic chemotherapy was notably higher in China (‘intermediate-risk’ n = 35; 22.9%/ ‘high-risk’ n = 33; 33.4%) compared with other countries (‘intermediate-risk range 2.7 − 8.9%/’high-risk’ range 5.4 − 12.8%). Intravesical chemotherapy was highest in China (‘intermediate-risk n = 35; 47.1%/’high-risk’ n = 33; 44.1%) compared with other countries (‘intermediate-risk range 15.4 − 38.4%/’high-risk’ range 13.6 − 37.2%) (Fig. 3b).

#### New and emerging treatments

**Fig. 4:**
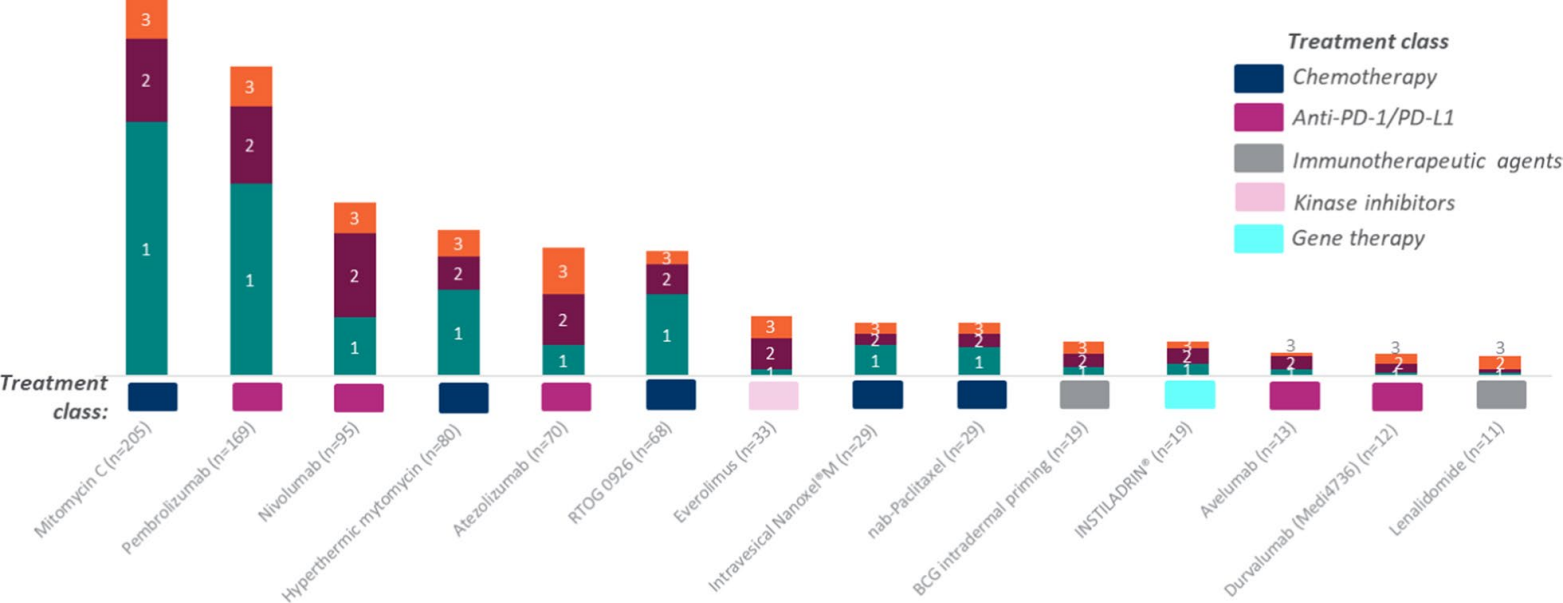
Top 3 ‘most impactful new therapies for BCG-unresponsive NMIBC patients’, as ranked by physicians (n = 508)

When considering treatments to recommend for BCG-unresponsive NMIBC patients (who are either medically unfit or refuse cystectomy), 247 (48.6%) physicians stated that they would recommend mitomycin C (MMC), 191 (37.6%) pembrolizumab, and 134 (26.4%) nivolumab. Physicians were also asked to rank the top 3 (in their opinion) ‘most impactful new therapies for BCG-unresponsive NMIBC patients’, assuming that all treatments were available to prescribe (i.e., including developmental products) (Fig. 4). Chemotherapy (in particular MMC) and PD-1 (pembrolizumab, nivolumab) and PD-L1 (atezolizumab) options were perceived by physicians as the most impactful novel treatment approaches for patients who have exhausted BCG treatment.

**Fig. 5:**
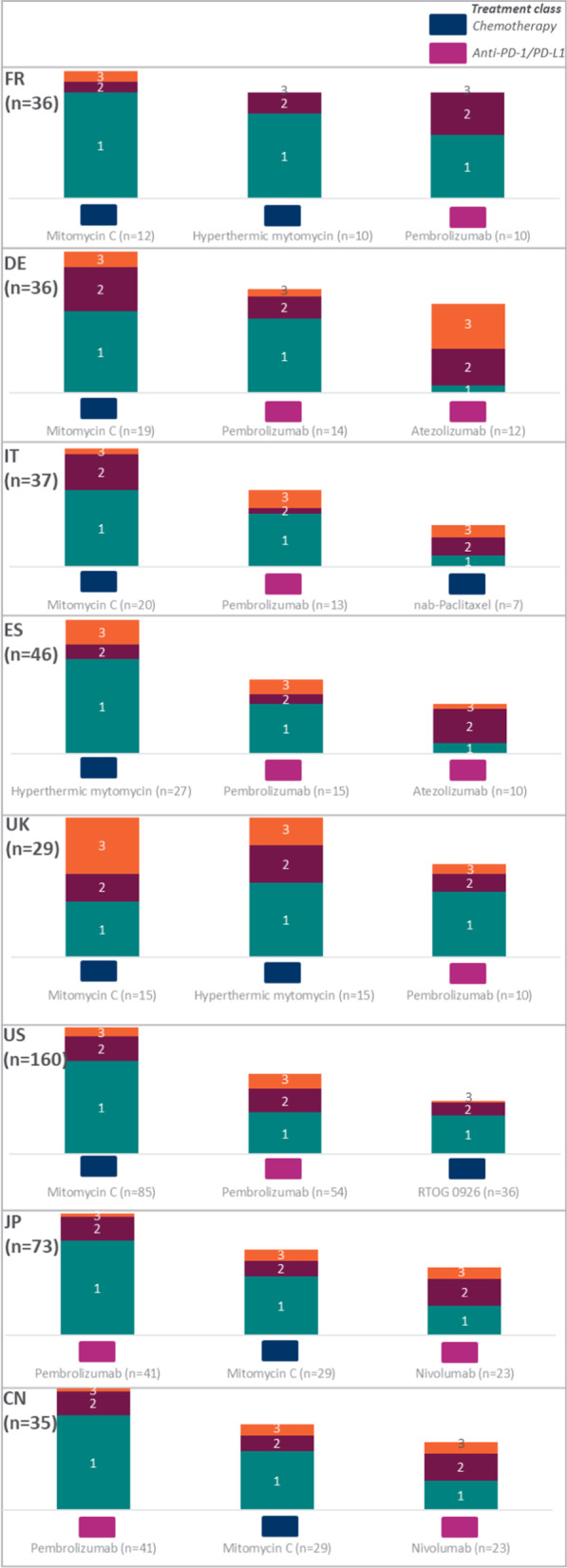
Country-specific top 3 ‘most impactful new therapies for BCG-unresponsive NMIBC patients’, as ranked by physicians

Country-specific analysis revealed a similar pattern to that observed at global level. MMC and pembrolizumab were reported in the top 3 for all countries except Spain, where hyperthermic mitomycin was reported instead of MMC. Nivolumab was ranked particularly high in the Asian countries (ranked in top 3 therapies for China and Japan). Chemotherapy therapies were most common (i.e., two out of top 3 ranked treatments were chemotherapy) in France, Italy, UK, and the US, whereas anti-PD-1/PD-L1 were most common in Germany, Spain, Japan, and China (i.e., two out of top 3 ranked therapies were anti-PD-1/PD-L1 agents) (Fig. 5).

## Discussion

There is currently a paucity of evidence in both the real-world and clinical trial setting on the management of BCG-unresponsive NMIBC patients. A recent systematic review identified only 23 trials suitable for inclusion in the meta-analysis investigating the efficacy and safety of current and emerging treatments (e.g. *Mycobacterium phlei* cell wall-nucleic acid complex, MMC, gemcitabine, pembrolizumab, nadofaragene firadenovec, and nab-paclitaxel) for NMIBC patients after treatment with BCG [13]. The pooled 12-month response rates were 24% for trials with ≥ 2 prior BCG courses (ranging from 9% with paclitaxel-hyaluronic acid to 37% with nadofaragene) and 36% for trials with ≥ 1 prior BCG courses (ranging from 10% with valrubicin to 48% with MMC in the ≥ 50% CIS subgroup, and from 47% with BCG + interferon to 62% with MMC in the ≤ 50% CIS subgroup). Most trials in the meta-analysis were single-arm, and r, Gill & Prasad (2020) have noted that this study design may be problematic in some cases, especially when evaluating costly therapies. For example, without evidence from randomized clinical trials comparing pembrolizumab with SOC in BCG-unresponsive NMIBC patients who are ineligible for or who have elected not to undergo cystectomy, itis not possible to determine the survival and QoL benefits of pembrolizumab, nor is it possible to determine its cost-effectiveness [18]. Nevertheless, emerging therapies currently in development show promising efficacy [13], and 3-year mid-term results in NMIBC patients who failed BCG and who were unwilling to undergo radical cystectomy have recently confirmed and extended short-term findings with intravesical gemcitabine as bladder-preserving treatment: disease-free survival was 33% at 36 months, while progression-free and overall survival were 65% and 67%, respectively[19].

The present real-world study surveyed physicians in eight countries, with the primary aim to understand the current patterns of treatment and disease management for the broad BCG-unresponsive NMIBC patient population, alongside collecting sufficient data on patients who do not undergo cystectomy. The most common tumor grading at diagnosis in the present study was CIS alone (34%), which is considerably higher than the 10% usually reported in the overall NMIBC population [20].

Use of biomarkers and treatment selection patterns in patients with BCG-unresponsive NMIBC in real-world clinical practice showed that almost one-quarter of physicians’ current NMIBC patient caseload was BCG-unresponsive, whereby BCG therapy was no longer considered an option. Just over one-third of physicians’ current NMIBC patient caseload had not received any BCG therapy, although this may have been because at the time of data collection, they were awaiting this treatment, following TURBT. It is also possible that the ongoing global BCG shortage may have played a role, as the worldwide shortage of BCG has limited the numbers of patients receiving adequate induction and maintenance, leading to higher recurrence and failure rates[21, 22]. However, this is unlikely to explain the high rate (66.8%) observed in Japan, where there is a relatively stable supply of the Tokyo 172 strain, manufactured and distributed in Japan [23].

Exploratory biomarker analyses areimportant to help identify patients most likely to respond to specific therapies or mechanisms of action and data from ongoing trials of novel immunotherapies in BCG-unresponsive NMIBC are awaited to determine therapy strategies that may optimize guidance for patient care [4]. In our real-world study, half of physicians did not regularly use biomarker tests in their practice, with particularly few physicians undertaking biomarker testing in Spain and Japan. If tests were conducted, the biomarker test that most physicians (almost one-third) used for surveillance was PD-1/PD-L1; of patients tested (28% of 2554 patients), over one-third were PD-L1 positive. PD-L1 positive expression was high in Asia (China and Japan), and anti-PD-1/PD-L1 treatments (pembrolizumab and nivolumab) were highly recommended for BCG-unresponsive NMIBC patients (medically unfit/refuse cystectomy) mostly by physicians in the Asia region, compared with physicians in Europe and the USA. China and Germany reported high PD-1/PD-L1 testing.

The same recommendation was observed for high-ranking treatment that physicians would recommend for BCG-unresponsive NMIBC patients (medically unfit/refuse cystectomy). Chemotherapy and anti-PD-L1 treatment options were considered impactful new therapies by 94.7% and 90.0% of physicians surveyed in this study, respectively.

The present study used the FDA definition for BCG-unresponsive disease. However, while this definition is useful for clinical trials, a recent study has reported that, in comparison with the less stringent European Association of Urology BCG-failure definition, the BCG-unresponsive definition may miss some patients who eventually progress: De Jong et al. (2020) found that 53/106 (50%) of the patients with progression were previously defined BCG-unresponsive, whereas 67/106 (63%) were once BCG-failures due to BCG-refractory disease or high-grade recurrences after BCG [24]. Hence it is possible that the potential NMIBC population with unmet need may be greater than apparent with a stringent definition of BCG-unresponsive disease.

In terms of the current management approach for the overall NMIBC patient population, physicians reported that TURBT and BCG were the most common current treatments received by their patients. Physicians reported that ‘high-risk’ patients were more likely to receive BCG treatment and systemic therapy, and twice as likely to receive radical cystectomy than ‘intermediate-risk’ patients. Given that our survey also included an enriched sample of patients who were BCG-unresponsive and did not undergo radical cystectomy, either because the patient was medically ineligible for a cystectomy (based on their physician’s assessment) or because they had refused cystectomy, the finding that, at global level, radical cystectomy use was limited in both risk groups, is not surprising. However, higher reports of this procedure were observed in the ‘high-risk’ group in Germany, Italy, Spain, and China. Overall, the use of partial cystectomy was even lower, although this, was notably higher in China compared with other countries.

The current study has several strengths and limitations: the study is retrospective in design, which naturally introduces potential biases; however, physicians were asked to supply information on the last 10 eligible patients according to the inclusion criteria, which should have minimised any patient selection bias and should have provided a diverse sample. Compared with randomized control trial populations, the present study included a more heterogenous sample, representing real-world clinical practice. Furthermore, this study evaluated a large patient sample across several different world regions. Requesting an equal number of patients from each site provides a wide selection of patients from a range of centres but does not consider the size of the patient pool at each site and therefore may introduce a level of bias based on the presenting population at smaller sites. To collect a robust representative sample of the specific patient population in a time-efficient manner, the study was designed to recruit a relatively large number of physicians to participate in the survey to minimise the impact of the clustering effect around a smaller number of sites. Only physicians who saw a minimum number of patients were included in the study, which may have excluded many physicians from taking part, but to achieve a robust sample of patients, it was necessary to select larger sites/treating physicians with large numbers of patients. Physicians’ responses to “most impactful treatment” (including developmental products) may be biased by prior experience of outcomes of products already available.

## Conclusion

The present study found that the most common treatments that patients received were TURBT and BCG. Given that emerging new treatments are driven by exploring biomarkers, it is interesting to note that in real-world clinical practice only half of physicians regularly tested their NMIBC patients for biomarkers, and wide variation was observed across countries, with particularly few physicians undertaking biomarker testing in Spain and Japan. However, PD-1/PD-L1 was the most common biomarker test used when such testing was undertaken (highest in China and Germany). Most physicians reported that, in addition to chemotherapy, anti-PD-L1 was an impactful new therapy.

## Supplementary Information

**Additional file 1.** Supplementary information regarding physician demographics and questions included in the survey.

## Data Availability

All data supporting the study is the intellectual property of Bristol Myers Squibb and can be made available on request.

## Abbreviations

BCG: Bacillus Calmette-Guérin;
BLCA-1: Human bladder cancer-associated nuclear matrix protein 1
BLCA-4: Human bladder cancer-associated nuclear matrix protein 4
BMI: Body mass index
BTA: Bladder tumor antigen
CEA: Carcinoembryonic antigen
cFH: Serum complement factor h
RTOG 0926: ChemoXRT for T1
CIS: Carcinoma in situ
CMP: Comprehensive metabolic panel
CN: China
CRF: Case report form
CT scan: Computerised tomography
DE: Germany
ECOG: Eastern Cooperative Oncology Group
EORTC: European Organization for Research and Treatment of Cancer
ES: Spain
FDA: Food and Drug Administration
FC: Fluorescent cystoscopy
FGFR3: Fibroblast growth factor receptor 3
FR: France
HRAS: HRAS proto-oncogene GTPase
IT: Italy
IVU: Intravenous urogram
JP: Japan
MIBC: Muscle invasive bladder cancer
MRI: Magnetic resonance imaging
MUM: Measurement of urine methylation
nab-Paclitaxel: Nanoparticle albumin-bound paclitaxel
NBI: Narrow band imaging
NMIBC: Non-muscle invasive bladder cancer
NMP22: Bladder protein check
PD-1/PD-L1: Programmed cell death protein 1/programmed death-ligand 1
PDD: Photodynamic diagnosis
PET: Positron emission tomography
PUB: Prostatic urethra biopsy
QoL: Quality of life
RPG: Retrograde pyelogram
SD: Standard deviation
SOC: Standard of care
TERT: Telomerase reverse transcriptase
Tp53: Tumor protein 53
TURBT: Transurethral resection of the bladder tumor
UK: United Kingdom
US: United States
WLC: White light cystoscopy
