# Supplementary material for: Multi-country clinical practice patterns, including use of biomarkers, among physicians’ treatment of BCG-unresponsive non-muscle invasive bladder cancer (NMIBC)
Source: BMC Urol. 2022 Feb 26;22:27. doi: 10.1186/s12894-022-00959-z (PMC8882282; doi:10.1186/s12894-022-00959-z)
Supplement: Supplementary file 1 — Additional file 1. Supplementary information regarding physician demographics and questions included in the survey. [file 12894_2022_959_MOESM1_ESM.docx]

**Supplementary Table 1. Physician demographics.**

| **Demographics** | **Overall n=508** | **France n=39** | **Germany n=39** | **Italy n=39** | **Spain n=49** | **UK n=36** | **USA n=180** | **Japan n=91** | **China**  **N=35** |
| --- | --- | --- | --- | --- | --- | --- | --- | --- | --- |
| **Age (years), mean (SD)** | 45.2 (8.9) | 44.5 (11.0) | 44.6  (6.1) | 46.6 (10.3) | 45.2 (8.7) | 46.8 (6.0) | 45.4  (9.8) | 44.4  (7.8) | 45.5 (8.1) |
| **Gender (male), n (%)** | 440  (86.6) | 32  (82.1) | 28  (71.8) | 27  (69.2) | 36  (73.5) | 32  (88.9) | 168  (93.3) | 91  (100.0) | 26 (74.3) |
| ***Clinical trials experience, n (%)*** | | | | | | | | | |
| Currently involved in NMIBC clinical trials | 57  (11.2) | 8  (20.5) | 3  (7.7) | 2  (5.1) | 7 (14.3) | 10 (27.8) | 15 (8.3) | 7  (7.7) | 5 (14.3) |
| Have been involved but not currently | 190 (37.4) | 13 (33.3) | 8  (20.5) | 18 (46.2) | 21 (42.9) | 21 (58.3) | 58 (32.2) | 31 (34.1) | 20 (57.1) |
| Have never been involved | 261 (51.4) | 18 (46.2) | 28  (71.8) | 19 (48.7) | 21 (42.9) | 5 (13.9) | 107 (59.4) | 53 (58.2) | 10 (28.6) |
| ***Proportion (%) of NMIBC practice time spent by physicians in following settings*** | | | | | | | | | |
| ***France (n=39)*, mean (SD)** | | | | | | | | | |
| University or regional hospital (CHU/CHR | 34.4 (47.2) |  |  |  |  |  |  |  |  |
| Private hospitals or clinic (centre privé) | 30.6  (45.0) |  |  |  |  |  |  |  |  |
| General hospital (CHG/CH) | 29.7 (44.7) |  |  |  |  |  |  |  |  |
| Specialized cancer hospital | 5.3  (22.3) |  |  |  |  |  |  |  |  |
| ***Germany (n=39)*, mean (SD)** | | | | | | | | | |
| University hospital/teaching hospital | 39.9  (48.1) |  |  |  |  |  |  |  |  |
| General hospital | 31.2 (43.6) |  |  |  |  |  |  |  |  |
| Specialist Cancer Center | 13.3 (33.7) |  |  |  |  |  |  |  |  |
| Hospital outpatient clinic | 13.1  (30.7) |  |  |  |  |  |  |  |  |
| Private oncologist/gynecologist center | 2.6 (16.0) |  |  |  |  |  |  |  |  |
| ***Italy (n=39)*, mean (SD)** | | | | | | | | | |
| Public general hospital (ospedale generale pubblico) | 46.4  (50.3) |  |  |  |  |  |  |  |  |
| Public teaching hospital (ospedale pubblico universitario) | 37.1 (47.7) |  |  |  |  |  |  |  |  |
| Cancer center (IRCCS/Istituti tumori) | 16.5 (36.3) |  |  |  |  |  |  |  |  |
| ***Spain (n=49)*, mean (SD)** | | | | | | | | | |
| Other academic/teaching hospital) | 77.2  (33.3) |  |  |  |  |  |  |  |  |
| Other non-teaching hospital | 12.6 (26.1) |  |  |  |  |  |  |  |  |
| Specialist cancer centre | 10.2 (23.9) |  |  |  |  |  |  |  |  |
| ***UK (n=36)*, mean (SD)** |  |  |  |  |  |  |  |  |  |
| Specialist cancer centre/tertiary referral treatment centre | 57.1  (40.6) |  |  |  |  |  |  |  |  |
| Other academic/teaching hospital | 30.0 (39.3) |  |  |  |  |  |  |  |  |
| Other non-teaching hospital | 8.9 (23.7) |  |  |  |  |  |  |  |  |
| Private hospital or clinic | 4.0  (6.6) |  |  |  |  |  |  |  |  |
| ***USA (n=180)*, mean (SD)** | | | | | | | | | |
| Community Practice | 51.8  (43.3) |  |  |  |  |  |  |  |  |
| Outpatient clinic | 18.3 (28.9) |  |  |  |  |  |  |  |  |
| Academic institution/not NCI cancer center | 15.0 (30.7) |  |  |  |  |  |  |  |  |
| Academic institution/NCI cancer center | 14.9  (31.3) |  |  |  |  |  |  |  |  |
| ***Japan (n=91)*, mean (SD)** | | | | | | | | | |
| Private hospital or clinic | 41.1  (46.2) |  |  |  |  |  |  |  |  |
| Other academic/teaching hospital | 28.8 (41.7) |  |  |  |  |  |  |  |  |
| Other non-teaching hospital | 19.3 (37.3) |  |  |  |  |  |  |  |  |
| Specialist cancer centre | 10.8  (28.1) |  |  |  |  |  |  |  |  |
| ***China (n=35)*, mean (SD)** | | | | | | | | | |
| Specialist cancer centre/tertiary referral treatment centre | 57.4  (46.3) |  |  |  |  |  |  |  |  |
| Other academic/teaching hospital | 38.3 (46.3) |  |  |  |  |  |  |  |  |
| Other non-teaching hospital | 4.1 (17.2) |  |  |  |  |  |  |  |  |
| Private hospital or clinic | 0.1  (0.9) |  |  |  |  |  |  |  |  |

SD: standard deviation.

**Supplementary Table 2. Survey questions.**

| **Section A: Physician demographics & caseload** |
| --- |
| AQ1. What is your age? |
| AQ2. What is your sex? |
| AQ3. Please select the region in which you provide care for your NMIBC patients? |
| AQ4. What proportion of your NMIBC practice time do you spend in each of the following settings? |
| AQ5. Which of the following best reflects your clinical trials experience in NMIBC? |
| AQ6. Which guidelines, if any, do you routinely follow when treating NMIBC patients? |
| AQ7. Please estimate i) your current total patient caseload (irrespective of condition, not just oncology), ii) your total bladder cancer patient caseload? |
| AQ8. Of your total bladder cancer caseload, how many patients do you estimate are i) muscle invasive bladder cancer (MIBC) patients ii) NMIBC patients? |
| AQ9. Of your total NMIBC patient caseload <n>, what percentage do you estimate is made up of i) patients currently treated with first course of Bacillus Calmette Guerin (BCG) ii) patients considered BCG unresponsive iii) patients not received any BCG treatment for NMIBC? *For definition of BCG-unresponsive criteria please click here* |
| AQ10. What proportion of your current NMIBC BCG unresponsive patients have the following tumour-risk classification? *For definition of risk classifications please click here* |
| AQ11. Of all the BCG unresponsive NMIBC patients you manage, what proportion were referred by another healthcare professional? *For definition of BCG-unresponsive criteria please click here* |
| AQ12. Of these referred patients, what proportion were referred by the healthcare professionals listed below? |
| **Section B: Physician management of NMIBC** |
| BQ1. Which of the following biomarkers, if any, do you regularly test for in NMIBC patients? |
| BQ2. Which of the following, if any, do you use to guide decisions on treatment of NMIBC patients? |
| **Section C: Physician awareness and use of treatment** |
| CQ1. What proportion of your current <i) intermediate and ii) high risk> NMIBC patients are currently receiving the following treatments/management approaches? *For definition of risk classifications please click here* |
| CQ2. Which of the following, if any, intravesical chemotherapies do you prescribe prior to BCG therapy for the following risk NMIBC patients? *For definition of risk classifications please click here* |
| CQ3ai. When suggesting BCG treatment in preference to radical cystectomy, which clinical aspects do you consider to be the most important? |
| CQ3aii. Of these, which <two/ three> do you consider to be the most important? Please assign rankings (1 to <2/3>, where 1 is “most important”) |
| CQ3bi. When suggesting radical cystectomy in preference to BCG treatment which clinical aspects do you consider to be the most important? |
| CQ3bii. Of these, which <two/ three> do you consider to be the most important? Please assign rankings (1 to <2/3> where 1 is “most important”) |
| CQ4. What methods do you use to determine if a patient is BCG unresponsive? For definition of BCG-unresponsive criteria please click here |
| CQ5. Of your BCG-unresponsive patients what percentage i) do not undergo cystectomy (medically unfit) ii) eligible and undergone/will undergo cystectomy iii) eligible and refused cystectomy *For definition of BCG-unresponsive criteria please click here* |
| CQ6a. When BCG treatment is no longer an option (patient will not be re-treated with BCG), which treatment/management approaches do you prescribe to the following risk level BCG unresponsive NMIBC patients? *For definition of risk classifications please click here* |
| CQ6b. When BCG treatment is no longer an option (patient will not be re-treated with BCG) which of the following, if any, intravesical or systemic chemotherapies do you prescribe to the following risk BCG-unresponsive NMIBC patients? *For definition of risk classifications please click here* |
| CQ7. Considering your current BCG-unresponsive patient caseload who do not undergo cystectomy, what proportion are ineligible due to the following reasons? *For definition of BCG-unresponsive criteria please click here* |
| CQ8. Considering your current BCG-unresponsive patient caseload who refused cystectomy, what proportion refused due to the following reasons? *For definition of BCG-unresponsive criteria please click here* |
| **Section D: Developmental products** |
| DQ1. Which of the following therapies for the treatment of BCG-unresponsive NMIBC patients are you a) aware of, b) would you recommend to BCG-unresponsive patients who are medically unfit for and/or refuse cystectomy? *Please click here for definition of ‘BCG-unresponsive’ patients* |
| DQ2. From your knowledge of prescribing, of the new therapies in development, which do you think will have the most impact on your prescribing for BCG-unresponsive NMIBC patients? *Please click here for definition of ‘BCG-unresponsive’ patients* *Please rank the top 3 with rank 1 being ‘most impact on my prescribing if available’, 2 being ‘second greatest impact’ and so on* |
| DQ3. Are you aware of any early /compassionate access treatment programmes for BCG-unresponsive NMIBC patients? |
| **Section E: Physician perceptions** |
| EQ1. To what extent do you agree with the following statements below? |
